# Estimation of Energy Intake by a Food Frequency Questionnaire: Calibration and Validation with the Doubly Labeled Water Method in Japanese Older People

**DOI:** 10.3390/nu11071546

**Published:** 2019-07-09

**Authors:** Daiki Watanabe, Hinako Nanri, Hiroyuki Sagayama, Tsukasa Yoshida, Aya Itoi, Miwa Yamaguchi, Keiichi Yokoyama, Yuya Watanabe, Chiho Goto, Naoyuki Ebine, Yasuki Higaki, Kazuko Ishikawa-Takata, Misaka Kimura, Yosuke Yamada, Kyoto-Kameoka Study Group

**Affiliations:** 1 National Institute of Health and Nutrition, National Institutes of Biomedical Innovation, Health and Nutrition, Tokyo 162-8636, Japan,; 2 Department of Pharmacology, St. Marianna University School of Medicine, Kanagawa 216-8511, Japan; 3 Faculty of Health and Sport Sciences, University of Tsukuba, Ibaraki 305-8577, Japan; 4 Laboratory of Applied Health Sciences, Kyoto Prefectural University of Medicine, Kyoto 602-8566, Japan; 5 Senior Citizen’s Welfare Section, Kameoka City Government, Kyoto 621-8501, Japan; 6 Institute for Active Health, Kyoto University of Advanced Science, Kyoto 621-8555, Japan; 7 Department of Health, Sports and Nutrition, Faculty of Health and Welfare, Kobe Women’s University, Hyogo 650-0046, Japan; 8 Faculty of Health and Sports Science, Doshisha University, Kyoto 610-0394, Japan; 9 Department of Health and Nutrition, Faculty of Health and Human Life, Nagoya Bunri University, Aichi 492-8520, Japan; 10 Faculty of Sports and Health Science, Fukuoka University, Fukuoka 814-0180, Japan

**Keywords:** food frequency questionnaire, energy intake, doubly labeled water, total energy expenditure, validity

## Abstract

Accurate assessments of a target population’s energy intake (EI) are essential to prevent poor nutritional status. However, self-reported dietary records (DRs) or food frequency questionnaires (FFQs) are not always accurate, thereby requiring validation and calibration studies. This study aimed to validate the EI estimated by a FFQ using the doubly labeled water (DLW) method. Participants were 109 Japanese older adults (50 women and 59 men) aged 65–88 years. The EI was obtained by a DR and 47-item FFQ over 1 year. The total energy expenditure (TEE) was measured by DLW for ~2 weeks. EI was significantly lower than TEE (*p* < 0.01); ratios of EI assessed by DR and FFQ against TEE were 0.91 ± 0.17 and 0.82 ± 0.22, respectively. TEE was significantly and moderately correlated with the EI estimated by the DR (*r* = 0.45, *p* < 0.01) and FFQ (*r* = 0.37, *p* < 0.01). Furthermore, the EI correlation coefficients estimated by DR and the FFQ in this study were not significantly different (*p* = 0.46). The EI/TEE ratio was significantly and negatively correlated with the body mass index (BMI). In conclusion, EI estimated with a DR or FFQ modestly correlated with TEE, and calibrating EI with a developed equation in this study can attenuate the underestimation of EI.

## 1. Introduction

Nutritional epidemiological studies are dependent on the accuracy of the habitual dietary survey results they use. The purpose of a dietary assessment is usually one of the following: (1) to compare the mean or median food intake between groups, (2) to rank individuals belonging to the same group, or (3) to evaluate an individual’s habitual food intake [1]. Epidemiological studies usually use food frequency questionnaires (FFQs), dietary records (DRs), or 24-hour dietary recalls (24 HRs) to assess a participant’s dietary habits. Of those, FFQs have been widely used in many epidemiological studies because they are easy to administer to more participants with a lower staff burden and cost than DRs or 24 HRs [2]. However, actual dietary intake has wide variations, which are dependent on food culture and dietary habits of the target population. To limit the burden of the participants, FFQs can include only a limited number of foods to maintain high feasibility. Thus, FFQs need validation studies to accurately and precisely estimate the dietary intake of the target population for these purposes [3].

In validation studies, energy and nutrient intake estimated by FFQs have been commonly compared with those estimated using DRs [4,5] or 24 HRs [6], which are considered more robust methods than FFQs. However, reporting biases associated with variables such as age [7,8,9], sex [10,11], body mass index (BMI) [8,9,11,12], smoking status [8,11], educational attainment [8,11], socioeconomic status [8,13], and social desirability [13] cannot be entirely avoided when evaluating the dietary intake using self-reporting dietary assessment methods such as DRs, 24 HRs, and FFQs. Misreporting can easily happen intentionally or unintentionally in self-reporting dietary assessment methods, which leads to systematical or random errors. In fact, it is known that these self-reporting dietary assessment methods do not accurately estimate an individual’s usual dietary intake [14,15,16,17]. Therefore, calibration with objective biomarkers is necessary to reduce reporting biases to a bare minimum [8,9,18].

For the energy intake (EI), when the body weight status is stable, the doubly labeled water (DLW) method is the preferred validation method when measuring the total energy expenditure (TEE) [7,8,9,10,12,15,16,19]. For example, a previous study has reported a comparison between the EI obtained by a FFQ and the TEE measured by the DLW method in Japanese middle-aged adults [19]. However, to the best of our knowledge, the validation of the EI estimated by FFQ using the DLW method in the Japanese older adult populations has not been addressed [20].

The population of people aged ≥65 years in Japan is rapidly increasing, being currently 35.6 million, a 28.1% increase as of 1 November 2018, and is even expected to increase by over 40% by 2055. Thus, epidemiological studies addressing health issues in the older population are critical to avoid a further increase in social burden and/or social security expenses. For that purpose, adequate tools assessing their food intake are required.

Our study group has launched an epidemiological study based on an older Japanese study, referred to as the Kyoto–Kameoka study. This study used a 47-item FFQ (in which one question is for alcohol) [5,21,22,23], which was originally developed and validated against DR for the middle-aged population [4,24]. This FFQ is currently being used in multi-site studies across the country, such as the Japan Multi-Institutional Collaborative Cohort (J-MICC) study [25]. Recently, we have validated the FFQ against DR in our cohort [5], but there are unresolved questions about the validity of FFQ against biomarkers like the DLW method, which does not rely on self-reporting tools.

In this study, we aimed to examine the validity of the EI estimated by the DR and 47-item FFQ against TEE measured by the DLW method and develop a calibration equation to address self-reporting bias for the EI estimated by FFQ.

## 2. Materials and Methods

### 2.1. Study Participants

The participants included a community-dwelling older population who lived in Kameoka city, Kyoto prefecture. The Kyoto–Kameoka study was a longitudinal cohort study, which conducted its first baseline survey on 29 July 2011. The details on this cohort have been described elsewhere [5,21,22,23,26,27,28]. Briefly, out of the 8319 participants who responded to the FFQ in the Kyoto–Kameoka study, 1379 participated in the additional face-to-face physical examination [5,26]. Of these participants, 147 individuals participated in the DLW study with a 7-day DR in May/June 2012 [5]. We excluded the participants who did not complete the 7-day DRs (*n* = 3) and had missing variables in the FFQ (*n* = 35). As a result, a total of 109 participants (50 women and 59 men) who completed both the FFQ and 7-day DR were included in the current study. The protocol of the Kyoto–Kameoka study was approved by the Ethics Committee of Kyoto Prefectural University of Medicine (RBMR-E-363) and by the National Institute of Health and Nutrition (NIHN187-3). Written informed consent was obtained from all participants, and the research was conducted in accordance with the Declaration of Helsinki.

Height and weight were measured using measurement apparatus combined with digital weight scale and stadiometer (DST-210S, Muratec-KDS Corp., Kyoto, Japan, with accuracy class 3 for weight in the Japanese Industrial Standards).

### 2.2. Dietary Assessment

The FFQ was administered using the questionnaires developed by Tokudome et al., which consists of 47 food and beverage items. The items inquired about year-round dietary habits [4,5]. Food and beverage consumption (except alcohol) frequencies were classified into eight categories: (1) never or seldom, (2) 1–3 times per month, (3) 1–2 times per week, (4) 3–4 times per week, (5) 5–6 times per week, (6) once per day, (7) twice per day, and (8) 3 or more times per day. For the item that inquired about alcohol consumption, the frequency and amount were recorded. This FFQ has been validated for middle-aged adults [4] and older adults aged ≥65 years [5], and it has been reported that the food and nutrition intake estimated using this FFQ was related to frailty [22,23] or oral health-related quality of life [21,28]. The FFQ was nested in the second baseline mail survey referred to as “*the Health and Nutrition Status Survey*”. All collected questionnaires were checked by three trained registered dietitians. The energy and nutrient intake was calculated taking into account the portion size, the frequency of appearance of each food and beverage category, and their weighted mean energy and nutrient value [29].

We collected DR over 7 consecutive days including both weekdays and weekends, during the DLW measurement period. The details of DR have been described before [5]. In brief, research staff were educated regarding how to administer the DR to participants. Each participant was provided a digital scale (TANITA, Tokyo, Japan), blank record sheets to record their DR, and paper media for education to record every item of food and beverage consumed daily.

### 2.3. Doubly Labeled Water (DLW)

The TEE was measured using the DLW method over ~2 weeks [30,31,32] during May and June 2012. On the first day (day 0), participants were summoned to our testing facilities in the morning, and they provided urine samples on site (baseline, BL). After providing BL urine samples, they were given a drink containing a premixed dose of 0.12 g/kg estimated total body water (TBW) of ^2^H_2_O (99.9 atom %, Taiyo Nippon Sanso, Tokyo, Japan) and 2.5 g/kg estimated TBW of H_2_^18^O (10.0 atom %, Taiyo Nippon Sanso, Tokyo, Japan) between 07:00 and 08:00. After drinking the beverage containing H_2_^18^O and ^2^H_2_O, urine samples were collected the next morning (day 1) and the mornings of days 2, 8, 9, 15, and 16. The urine samples were stored at −15 °C for later analysis by isotope ratio mass spectrometry (Hydra 20-20 Stable Isotope Mass Spectrometers; SerCon Ltd., Crewe, UK).

The corresponding references and each sample were measured in duplicates at the Fukuoka University Institute for Physical Activity (FUIPA). The detailed methodology implemented at FUIPA is described elsewhere [33,34]. CO_2_ was used as the equilibration gas for ^18^O measurements, and H_2_ was used for ^2^H measurements with a Pt catalyst. The ^18^O (No) and ^2^H (Nd) dilution spaces were determined by dividing by the dose of the administered tracer (as moles of ^2^H- or ^18^O-water) using the intercept method [30,31,32]. Because all of the ^18^O enrichment of the urine sample at day 16 were above 8‰, the intercept method was used with urine samples obtained at baseline and days 1, 2, 8, 9, 15, and 16. The Nd/No of the present study (1.036 ± 0.011, range 1.001–1.061) was similar to the value reported in a previous large-scale pooled analysis [35]. Thus, TBW (moL) was calculated as the mean of Nd (moL) divided by 1.041 and No (moL) divided by 1.007 [36]. We calculated carbon dioxide production rates (rCO_2_, moL/day) with the following equation: rCO_2_ = 0.455 × TBW × (1.007 ko − 1.041 kd), where TBW is the total body water and ko and kd are the rates of ^18^O and ^2^H elimination per day. The ko and kd were obtained using the modified two-point approach, and the slope between days 1 and 15 and that between days 2 and 16 were calculated and averaged.

TEE (kcal/day) was calculated using the modified Weir’s formula based on the rCO_2_ (moL/day) and 24-h estimated respiratory exchange ratio (RER) [37]: TEE (kcal/day) = 22.4 × (3.9 × (rCO_2_/RER) + 1.1 × rCO_2_), where 22.4 is the molar volume calculated from the dietary survey during the study period. The estimated RER was set at 0.86 for all participants, which was based on the previous study assessing the protein, fat, carbohydrate, and alcohol consumption (P/F/C/A) ratio of community-dwelling older people [31]. We assumed perfect nourishment balance conditions, which determine that food quotient (FQ) must be equal to the RER [38]. The quality control checklist of the DLW method is described in the International Atomic Energy Agency (IAEA) documents [39]. Fat-free mass (FFM) was calculated using TBW with the hydration factor of adults (0.732), as described in another IAEA document [39]. Fat mass (FM) was calculated as body weight minus FFM (kg).

### 2.4. Statistical Analysis

Covariate variables such as smoking status, educational attainment, living alone, socioeconomic status, and physical activity (going out once a week) were extracted from “*the Needs in the Sphere of Daily Life survey*” (first baseline survey) and “*the* *Health and Nutrition Status survey*” (second baseline survey), the details of which have been described elsewhere [5,21,22,23,26,27,28]. The BMI was calculated as body weight (kg) divided by the square of body height (m^2^). Before analysis, data distribution and normality (skewness and kurtosis) were checked. The continuous variables were shown as mean with standard deviation (SD) and were analyzed using the paired t-test. Ranges were also shown where appropriate. The categorical variables were shown as number with percentage. When missing answers or logical errors were identified, these values were handled as missing data. We previously reported that the accuracy and precision of the dietary intake estimated in the FFQ tend to be different according to age (<75 and ≥75 years) and sex groups [5]. Therefore, to confirm the accuracy and precision of the EI estimated by the DR and 47-item FFQ against those of the TEE measured by DLW, our statistical analyses were examined according to sex- (women and men) and age- (<75 and ≥75 years) stratified models. The accuracy of the mean EI was assessed by the EI/TEE ratio. The precision of ranking an individual’s EI was evaluated by Pearson’s correlation and Spearman’s rank correlation analysis between the TEE and EI estimated by each dietary assessment. To compare the correlation coefficient between the EI estimated by the DR and FFQ against TEE, we investigated whether the participant had an equivalent accuracy using the equation described by Meng et al. [40]. To develop an equation to calibrate the EI estimated by the FFQ using TEE, we conducted a multiple regression analysis with stepwise methods. In this analysis, the variables were age (<75 or ≥75 years), sex (woman or man), BMI (continuous variable), smoking status (never smoker, past smoker, and current smoker), educational attainment (<13 or ≥13 years), living alone (yes or no), socioeconomic status (high or low), physical activity (going out once a week, yes or no), and EI estimated by FFQ (continuous variable). We developed an equation to calibrate the EI estimated by this FFQ using the multiple regression analysis, the details of which were described in the results. The association between BMI groups (<18.5, 18.5–24.9, or ≥25.0 kg/m^2^) and EI/TEE was shown as median with interquartile range (IQR) and was analyzed using the Jonckheere–Terpstra trend test. A *p* value of 0.05 from two-sided tests was considered significant. The statistical analyses were performed with the use of STATA MP, version 15.0 (StataCorp LP, College Station, TX, USA).

## 3. Results

The characteristics of the participants in the present study are shown in Table 1. The mean age was 72.2 (range: 65 to 84) and 73.5 (range: 66 to 88) years in women and men, respectively. The mean BMI of men was 23.0 (range: 16.8 to 31.1) and 22.7 (range: 14.3 to 30.0) kg/m^2^ in women and men, respectively. The mean FFM was 32.8 and 43.9 kg in women and men, respectively. Notably, a low BMI (<18.5 kg/m^2^) was found in 5 (10.0%) women and 5 (8.5%) men, while 14 (28.0%) women and 13 (22.0%) men were found to be overweight (≥25.0 kg/m^2^). In addition, we found that 46 (81.6%) women and 57 (94.9%) men went out once a week and that 13 (26.0%) women and 22 (37.3%) men had high educational attainment (≥13 years).

Table 2 shows the comparison of TEE measured by DLW and EIs assessed with DR and FFQ. Among all participants, the mean TEE was 2175 (range: 1246 to 3435) kcal/day, and the mean EI estimated from the DR and 47-item FFQ were 1972 (range: 1306 to 2948) and 1774 (range: 736 to 3461) kcal/day, respectively. The ratios of EI assessed with the DR and 47-item FFQ to TEE measured by DLW were 0.91 (range: 0.57 to 1.52) and 0.82 (range: 0.20 to 1.61). The ratio of EI estimated by FFQ to TEE measured by DLW were significantly lower than ratio of EI estimated by DR to TEE (−0.09, 95% confidence interval: −0.13 to −0.05, *p* <0.001). We observed similar results in the stratified groups, except in participants aged ≥75 years old.

The correlation coefficients of TEE measured by DLW and of EI assessed with the DR and 47-item FFQ are shown in Table 3. Pearson’s and Spearman’s correlation coefficients of EI estimated by DR and this FFQ were significantly correlated with TEE measured by DLW. Moreover, there was no significant difference in the correlation coefficient between EI estimated by DR and this FFQ against TEE using Meng’s Z-test comparison *(p* values are shown in the right column). We observed similar results in the stratified groups except in women.

Table 4 shows the results of the multivariate analysis of the linear model with TEE measured by DLW as the dependent variable and the EI estimated by this FFQ as the explanatory variable. The determinant coefficient (R^2^) of the linear regression analysis was 0.36 for this FFQ. The age, sex, BMI, and EI estimated by this FFQ were included as significant independent variables in the model, while smoking, education, living, socioeconomic status, and physical activity (going out once a week) were not included. We developed an equation to calibrate the EI estimated by this FFQ using the multiple regression analysis. The models followed the equation:

ε = *β*_0_ + *β*_1_ age_1_ + *β*_2_ sex_2_ + *β*_3_ BMI_3_ + *β*_4_ EI_4_ (1)

This equation is modeled to calibrate the EI estimated by this FFQ, where ε is the calibrated mean EI and the intercept (*β*_0_) is 1384.92 kcal in this FFQ. For binary variables, the coefficient of age (*β*_1_) was −166.98 kcal for ≥75 years, and the coefficient for sex (*β*_2_) was −354.72 kcal for women. For continuous variables, the coefficient for BMI (*β*_3_) was 25.55 kcal/[kg/m^2^], and the coefficient for EI (*β*_4_) was 0.24 kcal with the 47-item FFQ. All regression coefficients are shown in Table 4.

A review of previous and current studies on the developed calibration equation for energy intake from a self-reported dietary assessment and DLW is shown in Table 5. Our results were similar to those of previous studies wherein the age, BMI, and EI estimated by FFQ were determined as significant independent variables in the calibration equation.

The comparison of the EI/TEE ratios according to the BMI group is shown in Table 6. We calculated calibrated EI values using the equation (1) that we created based on our multivariate analysis (Table 4). In addition, we determined EI/TEE from both uncalibrated and calibrated EI values divided by TEE measured by DLW. Using the Jonckheere-Terpstra trend test, we found no significant differences in the uncalibrated EI/TEE between the different BMI groups or between all types of dietary assessment. However, we observed that the higher BMI group tended to have lower EI/TEE ratios than the lower BMI group. Pearson’s correlation coefficient indicated that there was a significant correlation between BMIs and uncalibrated EI/TEEs (correlation coefficient; −0.19, *p* = 0.048) for DR. However, there was no significant correlation between BMI and EI/TEE according to the two types of correlation analysis, and higher BMI tended to be associated with a negative EI/TEE in all uncalibrated dietary assessment methods. The calibrated EI/TEE ratios had lower correlation coefficients than the uncalibrated EI/TEE ratios in FFQ.

## 4. Discussion

We have recently reported the validation of this 47-item FFQ against the 7-day DR in older adults [5]. However, even in DR, self-reporting methods have large potential error and bias. Therefore, we aimed to evaluate the precision and accuracy of EI values estimated by this FFQ against TEE measured by the DLW method in the current study. We have shown that EI estimated by FFQ correlates modestly with TEE measured by DLW. In addition, this study suggests that EI underreporting may be attenuated by our developed calibration equation based on our multiple regression analysis, which included variables affecting self-reporting biases in overweight individuals.

We have shown that EI/TEE ratios estimated using the FFQ are lower than those estimated by DR (Table 2). Moreover, it has been reported that, in comparison with longer questionnaires and DR, short dietary survey questionnaires tend to underestimate the energy and nutrient intake more [41]. However, a previous study has reported the development of several FFQs to assess the habitual Japanese dietary intake, where the median number of food items was 45 (ranging from 9 to 169) [20]. The number of food items in our FFQ was similar to this median. In addition, our FFQ was developed based on foods that contribute to 85% of the between-person variance for each energy and nutrient intake in the middle-aged population [24]. The reason for the underestimation may be that the foods and beverages listed in the short questionnaire may not sufficiently reflect those habitually consumed by the target population. However, the current studies had a higher EI/TEE estimated by FFQ and DLW than that in the previous studies (Table 5) [8,9]. These differences may not be explained due to the differences in the number of food items included in FFQs, but may be because of the differences in sex, race/ethnicity, and BMI. Therefore, these population attributes need to be considered when comparing studies reporting EI by FFQ. For the individual ranking, the EI estimated by DR and FFQ correlated modestly with the TEE measured by DLW (Table 3). Interestingly, we found no significant correlation between EI and TEE with any of the EI assessment methods in women. A previous study has reported that, among middle-aged Japanese adults, women tend to demonstrate a weaker correlation between EI and TEE than men [19]. Both studies in Table 5 included only women, while, in the current study, 54% of the participants were men, which perhaps explains the higher EI/TEE of the current study than the studies presented in Table 5.

In addition, there was a significant difference between the ratio of EI estimated by DR to TEE and that estimated by FFQ to TEE in participants aged <75 years old but not ≥75 years old (Table 2). Moreover, participants aged ≥75 years old tended to have a higher correlation between TEE measured by DLW and EI assessed by FFQ than participants aged <75 years old (Table 3). We previously reported that the number of food items consumed daily tended to decrease in older people [5]. Therefore, we speculated that a lower number of food items may be sufficient to estimate the energy and nutrient intake using an FFQ in older populations, as the number of food items affecting inter-individual variability consumed by older people seemed smaller than that of middle-aged adults. This is perhaps the reason why, even though the FFQ has limited food items compared with the DR, there was no difference in the results obtained from DR and FFQ in the present study.

The EI estimated by self-reporting dietary assessment methods has been reported to be associated with variables such as age [7,8,9], sex [10,11], BMI [8,9,11,12], smoking status [8,11], educational attainment [8,11], socioeconomic status [8,13], and social desirability [13], all of which can generate reporting bias. We included these variables in our multiple regression analysis and showed that age, sex, BMI, and EI were related to TEE measured by DLW (Table 4). Previous studies reported that the reporting bias of dietary intake error was associated with BMI, age, race/ethnicity, annual income, physical activity, and diet change intervention (Table 5). Some epidemiological studies have reported that, for individuals with a high BMI, the EI calculated using self-reporting dietary assessment methods tends to be underestimated [8,9,11,12]. We have shown that there tends to be a negative (but not quite significant) correlation between BMI and EI/TEE calculated using an uncalibrated EI estimated by FFQ (Table 6). Notably, this error may not only decrease the accuracy of the group’s mean EI but also induce the misclassification of individual ranking. This makes EI estimated by self-reporting dietary assessment methods difficult to use in nutritional epidemiological studies. Previous works have reported that calibrating EI using TEE measured by the DLW method using multiple regression analysis improved the measurement error from self-reporting dietary assessment methods [8,9]. Similarly, we have also shown that calibration lowers the correlation coefficient between BMI and EI/TEE (Table 6). Recently, some nutritional epidemiological studies have reported that EI is associated with the incidence of diabetes [42] and cancer [43] only when EI is calibrated. Based on these reports, we could speculate that the calibration of EI attenuates measurement errors due to reporting bias. Therefore, this approach is helpful to assess the relationship between EI and disease in nutritional epidemiological studies because the self-reporting bias problem in EI assessment makes it unsuitable for this type of study.

Our study has several limitations. First, we assumed perfect nourishment balance conditions in all participants to be able to use the DLW method. However, since we did not confirm this assumption, it is possible that not every participant had a perfect nourishment balance. Second, we could not develop a calibration equation of macronutrients by other biomarkers including urinary samples and serum concentrations. It is a definite limitation of the study that the calibration is only useful for studies focused on energy intake, but not useful for studies that are focused on the role of a specific macronutrient in the incidence or prevalence of disease. Third, there was an interval of 2–3 months between the TEE measurement by the DLW method and the survey with FFQ. In addition, we could not follow a multipoint method for the measurement of TEE by the DLW method. These experimental conditions might have led to random and systematic errors because participants may have modified their usual activity. Finally, this study only included participants who consented to participate in the physical check-up examination in the Kyoto–Kameoka study. Such participants might be more health conscious than those who did not. In addition, this study included a smaller number of participants than in previous studies (Table 5). Therefore, the distribution of EI and TEE may not reflect that of the general population because these participants were not randomly sampled from the general Japanese older population.

DR faces the problem of measurement error, and there is also a large burden on the participant. However, in our study, in comparison with the measured TEE by the DLW method, the energy intake estimated from DR tended to have a higher correlation coefficient than that from FFQ. Therefore, DR may invariably be important in nutritional epidemiology. Better-quality studies will help assess the precision and accuracy of energy intake estimated using recovery biomarkers such as DLW. Energy intake estimated by self-reporting dietary assessment methods including FFQ tends to be underestimated. The calibration technique using DLW for energy intake estimated by FFQ may be helpful to assess the relationship between EI and disease in nutritional epidemiological studies.

## 5. Conclusions

This study suggests that EI estimated with a FFQ is modestly correlated with TEE and that our developed calibration equation may attenuate the effect of self-reporting biases in the estimation of EIs by FFQs in Japanese older adults. This approach could be further our understanding of the relation between EI and disease risk in Japanese older adults, while substantially addressing measurement error problems that have long plagued the field of nutritional epidemiology.

## Figures and Tables

**Table 1:** Characteristics of study participants according to sex group.

	Women (*n* = 50)			Men (*n* = 59)		
	Mean		SD or %	Mean		SD or %
Age (years) ^a^	72.2	±	4.6	73.5	±	6.0
≥75 years ^b^	16	(32.0)		23	(39.0)	
Height (cm) ^a^	151	±	5	165	±	5
Body weight (kg) ^a^	52.2	±	7.8	61.6	±	8.6
BMI (kg/m^2^) ^a,c^	23.0	±	3.5	22.7	±	2.8
<18.5 ^b^	5	(10.0)		5	(8.5)	
18.5-24.9 ^b^	31	(62.0)		41	(69.5)	
≥25.0 ^b^	14	(28.0)		13	(22.0)	
Fat free mass (kg) ^a^	32.8	±	3.5	43.9	±	5.0
Smoking status ^b,d^						
Never smoker	25	(50.0)		36	(61.0)	
Past smoker	21	(42.0)		19	(32.2)	
Current smoker	3	(6.0)		1	(1.7)	
Educational attainment ^b,e^						
<13 years	33	(66.0)		35	(59.3)	
≥13 years	13	(26.0)		22	(37.3)	
Living alone ^b,f^	10	(20.4)		3	(5.1)	
Socioeconomic status ^b,g^						
Low	24	(48.0)		30	(50.8)	
High	24	(48.0)		28	(47.5)	
Once a week go out ^b,h^	46	(92.0)		57	(96.6)	
Caregiving ^b^	0	(0.0)		0	(0.0)	

Abbreviations: BMI: body mass index; SD: standard deviation. ^a^ Continuous values are shown as mean ± standard deviation. ^b^ Categorical values are shown as number (percentage). ^c^ BMI was calculated as body weight in kilograms divided by the square of height in meters (kg/m^2^). ^d^ Missing; women (*n* = 1) and men (*n* = 3). ^e^ Missing; women (*n* = 4) and men (*n* = 2). ^f^ Missing; women (*n* = 1). ^g^ Missing; women (*n* = 2) and men (*n* = 1). ^h^ Missing; women (*n* = 4) and men (*n* = 2).

**Table 2:** Comparison of the total energy expenditure measured by doubly labeled water and energy intake assessed with dietary record and food frequency questionnaire.

	TEE (kcal/day)	Energy Intake (kcal/day)		Ratio of EI Estimated by DR to TEE	Ratio of EI Estimated by FFQ to TEE	Mean Difference ^b^	*p* Value ^c^
	DLW	7-day DR	47 Items FFQ ^a^				
Total (*n* = 109)	2175±420	1972±299	1774±483	0.91±0.17	0.82±0.22	−0.09 (−0.13 to −0.05)	**<0.001**
Sex							
Women (*n* = 50)	1955±284	1815±205	1619±341	0.93±0.17	0.83±0.24	−0.10 (−0.15 to −0.05)	**<0.001**
Men (*n* = 59)	2368±430	2105±302	1905±546	0.89±0.17	0.80±0.20	−0.09 (−0.15 to −0.03)	**0.007**
Age							
<75 years (*n* = 70)	2209±393	1993±321	1718±493	0.90±0.15	0.78±0.23	−0.12 (−0.18 to −0.07)	**<0.001**
≥75 years (*n* = 39)	2114±463	1936±254	1874±454	0.92±0.19	0.89±0.20	−0.03 (−0.10 to 0.02)	0.14

Abbreviations: CI: confidence interval; DLW: doubly labeled water; DR: dietary record; FFQ: food frequency questionnaire; SD: standard deviation; TEE: total energy expenditure. The mean ± SD or mean difference (95% confidence interval (CI)) was also shown. ^a^ Assessed using the 47-item FFQ previously developed and validated by Tokudome et al. [4,5]. ^b^ The mean difference was determined by subtracting the ratio of energy intake (EI) estimated by DR to TEE from that estimated by FFQ to TEE. ^c^ To compare the accuracy of the energy intake, statistical analysis was conducted using paired t-test to compare the ratio of EI estimated by DR to TEE with that estimated by FFQ to TEE. Bold values are statistically significant (*p* < 0.05) and indicate DR and FFQ are not equivalently accurate as compared to TEE.

**Table 3:** Correlation coefficients of total energy expenditure measured by doubly labeled water and energy intake assessed with dietary record and a food frequency questionnaire.

	Energy Intake				*p* Value ^b^
	7-Day DR		47-Item FFQ ^a^		
**Total (*n* = 109)**					
Pearson’s correlation coefficient	0.45	^**^	0.37	^**^	0.46
Spearman correlation coefficient	0.46	^**^	0.39	^**^	0.46
Sex stratified model					
Women (*n* = 50)					
Pearson’s correlation coefficient	0.09		0.19		0.57
Spearman’s correlation coefficient	0.08		0.12		0.81
Men (*n* = 59)					
Pearson’s correlation coefficient	0.35	^**^	0.30	^*^	0.78
Spearman’s correlation coefficient	0.34	^**^	0.33	^*^	0.95
Age stratified model					
<75 years (*n* = 70)					
Pearson’s correlation coefficient	0.50	^**^	0.32	^**^	0.17
Spearman’s correlation coefficient	0.51	^**^	0.36	^**^	0.17
≥75 years (*n* = 39)					
Pearson’s correlation coefficient	0.36	^*^	0.54	^**^	0.22
Spearman’s correlation coefficient	0.40	^*^	0.51	^**^	0.45

Abbreviations: DR: dietary record, FFQ: food frequency questionnaire. ^a^ Assessed using the 47-item FFQ previously developed and validated by Tokudome et al. ^b^ Comparison of correlation coefficients between DR and the 47-item FFQ (Tokudome et al. [4,5]) (calculation according to Meng et al. [40]). Bold values are statistically significant (*p* < 0.05). If the results presented no significant difference, these relationships were considered to be of equivalent precision in terms of energy intake estimated by DR and FFQ. * Correlation coefficients between two methods: *p* < 0.05 (compared with TEE). ** Correlation coefficients between two methods: *p* < 0.01 (compared with TEE).

**Table 4:** Stepwise multiple regression analysis with the total energy expenditure measured by doubly labeled water as a dependent variable.

	47-Item FFQ (R^2^ = 0.36) ^a^				
	RC	SE	β	*p* Value	Collinearity VIF
Intercept	1384.92	276.29	0	<0.001	
Age (reference, ≥75 years)	−166.98	69.35	0.192	0.018	1.03
Sex (reference, women)	−354.72	69.02	0.423	<0.001	1.10
BMI	25.55	10.47	0.192	0.016	1.00
Energy intake ^b^	0.24	0.07	0.278	0.001	1.12

Abbreviations: BMI: body mass index; FFQ: food frequency questionnaire; RC: regression coefficient; SE: standard error; VIF: variance inflation factor. ^a^ Assessed using the 47-item FFQ previously developed and validated by Tokudome et al. [4,5]. ^b^ Estimated by the corresponding FFQ.

**Table 5:** Review of previous and current studies on the developed calibration equation for energy intake from a self-reported dietary assessment and doubly labeled water.

First Author, Year [Reference No.] (Study)	No. of Participants (Sex, % Men)	Mean Age, Years (SD)	Mean BMI, kg/m^2^ (SD)	FFQs		24 HRs or DRs		DLW	EI/TEE Ratio		Calibration Equation in FFQs	
				Type of FFQ (No. of Food Items)	Mean Intake, kcal/day	Type of Method (No. of Assessments)	Mean Intake, kcal/day	TEE, kcal/day	FFQ/DLW	DR or 24HR/DLW	R^2^	Adjusted Factors ^a^
Neuhouser, 2008 [8] (NBS)	544 (0)	70.9 (6.3)	28.2 (5.5)	WHI (122-item)	1443 ^b^	AMPM with NDSR ^c^ (2-day)	N/A	2056 ^b^	0.70 ^b^	N/A	0.31	Energy intake (FFQ), BMI, age, race/ethnicity, annual income, physical activity, diet change intervention
Prentice, 2011 [9] (NPAAS)	450 (0)	70.5 (6.0)	28.5 (6.4)	WHI (122-item)	1455	DR (4-day)	1617	2023	0.72	0.80	0.42	Energy intake (FFQ), BMI, age, race/ethnicity
Current study (Kyoto–Kameoka study)	109 (54.1)	72.9 (5.4)	22.9 (3.2)	Tokudome et al ^d^ (47-item)	1774 ^d^	DR (7-day)	1972	2175	0.82 ^d^	0.91	0.36 ^d^	Energy intake (FFQ), BMI, age, sex

Abbreviations: AMPM: Automated Multiple-Pass Method validation study; BMI: body mass index; DLW: doubly labeled water; FFQ: food frequency questionnaire; N/A: not available; NBS: Nutrition Biomarker Study; NPAAS: Nutrition and Physical Activity Assessment Study; NDSR: Nutrition Data System for Research; SD: standard deviation; TEE: total energy expenditure; WHI: Women’s Health Initiative; 24 HR: 24-hour dietary recall. ^a^ The factors needed for the calibration equation for energy intake estimated by FFQs. ^b^ The value was calculated by weighted mean for intervention and comparison groups. ^c^ Software from the University of Minnesota Nutrition Coordinating Center (Minneapolis, Minnesota). ^d^ The FFQ from Tokudome et al. has been validated [4,5].

**Table 6:** Comparison of the ratio of energy intake to total energy expenditure according to body mass index groups.

EI/TEE ^a^	BMI (kg/m^2^)						*p* for Trend ^b^	Correlation Coefficient		
	< 18.5 (*n* = 10)		18.5 to 24.9 (*n* = 72)		≥ 25.0 (*n* = 27)			Pearson’s		Spearman’s
Dietary record										
Uncalibrated ^c^	1.02	(0.96 to 1.13)	0.92	(0.78 to 1.02)	0.92	(0.79 to 1.06)	0.30	−0.19	^*^	−0.18
47-item FFQ ^d^										
Uncalibrated ^c^	0.84	(0.81 to 0.98)	0.85	(0.75 to 0.96)	0.80	(0.60 to 0.95)	0.12	−0.12		−0.17
Calibrated ^e^	1.07	(0.95 to 1.12)	0.99	(0.91 to 1.08)	1.03	(0.89 to 1.11)	0.66	−0.01		−0.01

Abbreviations: FFQ: food frequency questionnaire; TEE: total energy expenditure. The values are shown as median (interquartile range) or correlation coefficient. ^a^ Energy intake was assessed by 7-day DR and 47-item FFQ. TEE was measured by the DLW method. The EI/TEE was calculated using the estimated energy intake from each dietary assessment divided by TEE. ^b^ Statistical analysis was performed using a Jonckheere-Terpstra trend test. ^c^ Crude EI value estimated from each dietary assessment. ^d^ Assessed using the 47-item FFQ previously developed and validated by Tokudome et al. [4,5]. ^e^ Calibrated value by our developed equation (Table 4). * Correlation coefficients between BMI (as a continuous value) and EI/TEE (*p* < 0.05).
